# Alternations of NF-κB Signaling by Natural Compounds in Muscle-Derived Cancers

**DOI:** 10.3390/ijms241511900

**Published:** 2023-07-25

**Authors:** Justyna Radzka, Zofia Łapińska, Urszula Szwedowicz, Agnieszka Gajewska-Naryniecka, Agnieszka Gizak, Julita Kulbacka

**Affiliations:** 1 Department of Molecular Physiology and Neurobiology, Faculty of Biology, University of Wroclaw, 50-335 Wroclaw, Poland; justyna.radzka@uwr.edu.pl (J.R.); agnieszka.gizak@uwr.edu.pl (A.G.); 2 Department of Molecular and Cellular Biology, Faculty of Pharmacy, Wroclaw Medical University, 50-556 Wroclaw, Poland; zofia.lapinska@student.umw.edu.pl (Z.Ł.); urszula.szwedowicz@student.umw.edu.pl (U.S.); agnieszka.gajewska-naryniecka@umw.edu.pl (A.G.-N.); 3 Department of Immunology, State Research Institute Centre for Innovative Medicine, 08410 Vilnius, Lithuania

**Keywords:** muscle cancers, NF-κB, curcumin, catechin, resveratrol, quercetin, berberine, CAPE, cucurbitacin E

## Abstract

The NF-κB-signaling pathway plays a crucial role in cancer progression, including muscle-derived cancers such as rhabdomyosarcoma or sarcoma. Several natural compounds have been studied for their ability to alter NF-κB signaling in these types of cancers. This review paper summarizes the current knowledge on the effects of natural compounds, including curcumin, resveratrol, quercetin, epigallocatechin-3-gallate, and berberine, on NF-κB signaling in muscle-derived cancers. These compounds have been shown to inhibit NF-κB signaling in rhabdomyosarcoma cells through various mechanisms, such as inhibiting the activation of the IKK complex and the NF-κB transcription factor. These findings suggest that natural compounds could be potential therapeutic agents for muscle-derived cancers. However, further research is needed to fully understand their mechanisms of action and potential clinical applications.

## 1. The Role of NF-κB in Carcinogenesis

The NF-κB factor has been shown to be responsible for regulating genes involved in tumor promotion (e.g., cell proliferation, angiogenesis, or adhesion). In addition, the activation of the NF-κB factor affects the production of prostaglandins through the COX2 gene, which is overexpressed in various types of cancer [1]. In neoplastic tissues, where an elevated level of the NF-κB factor is observed, pro-inflammatory cytokines are accumulated, which influences the formation of a pro-neoplastic environment. Long-term inflammation results in genomic instability and the appearance of genetic mutations that support the formation and development of tumors [2]. Apoptotic genes such as the *FLICE* inhibitory protein, survivin, *XIAP*, the *c-IAP1*/*2* inhibitor, and the *Bcl*-*2* group of proteins are overexpressed in many types of cancer cells, and the NF-κB factor is responsible for inducing these genes. Therefore, it is believed that NF-κB controls anti-apoptotic mechanisms in tumorigenesis [3]. Studies performed on human prostate cancer cells (cell line PC-3M) show that NF-κB promotes angiogenesis, invasion, and metastasis by regulating vascular endothelial growth factor (VEGF) and matrix metalloproteinase (MMP) [4]. It has been proven that the NF-κB factor affects resistance to various chemotherapeutic agents. NF-κB is involved in the modification of *mdr1* gene expression, the product of which is P-glycoprotein (P-gp). This product is a plasma membrane-associated multidrug transporter that affects the outflow of chemotherapy drugs and contributes to resistance to treatment [5]. Studies have shown that NF-κB inhibits P-glycoprotein expression and may reactivate chemosensitivity [6]. Studies using cell cultures and in vivo studies on laboratory animals revealed that the NF-κB factor is involved in the degradation of muscle proteins [7]. In the study by Moore–Carrasco et al., rats with hepatocellular carcinoma (cell line AH-130) were treated with the NF-κB inhibitor (SP100030) and the activator protein-1 (AP-1). This treatment was proved to be effective in the treatment of skeletal muscle atrophy [8]. Muscle wasting accompanies aging and a pathological condition, such as cancer. A study using a murine model suggested that NF-κB levels in skeletal muscle wasting tissues were elevated in animals with LLC (Lewis Lung Cancer). The study showed that the inhibition of NF-κB activity by transgenic overexpression of the super-repressor mutant protein IκBα (i.e., IκBαSR) significantly attenuated skeletal muscle and body weight loss in mice with LCC [9].

## 2. Muscle and the Transcription Factor NF-κB

Nuclear factor NF-κB is the main transcription factor with a number of roles in all mammalian cells, e.g., the production of cytokines or stimulating DNA transcription. It is also involved in cell reactions to stimuli such as UV radiation, stress, or heavy metals. Since the discovery of the NF-κB factor, it has gained a lot of attention in the scientific world due to its wide involvement in various biological processes [10]. In recent years, the potential involvement of the NF-κB pathway in muscle development has begun to emerge.

### 2.1. Stages of Myogenesis

**Figure 1:**
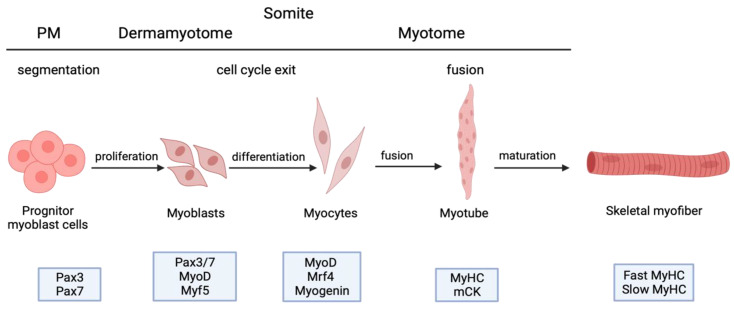
This diagram illustrates the process of paraxial mesoderm (PM) developing into skeletal muscle, showing the developmental sequence (**top**) and intermediate cell types with their corresponding marker genes (**bottom**) from left to right [15]. Created with BioRender.com.

Myogenesis begins in embryonic development in the paraxial mesoderm. The mesoderm forms somites, which divide into the dermomyotome and sclerotome. The dermomyotome further divides into the dermatome and myotome. Stimulated by environmental signals, myogenic precursor cells undergo an epithelial–mesenchymal transition (EMT), migrate to limb buds, and form the myotome. The early myotome comprises primary myocytes, the embryo’s first postmitotic skeletal muscle cells. Mononucleated myocytes undergo several stages of proliferation, differentiation, and fusion, leading to the development of mature multinucleated myofibers that are essential for muscle function. Migrating somites are characterized by two pairs of homeodomain transcription factors, Pax3 and Pax7 expression, that need to be downregulated for the myogenic program to proceed [11]. A minor portion of migrating somites generates satellite cells that subsequently reside between the sarcolemma and basal lamina of myofibers [12]. The differentiation of skeletal muscles is controlled via the basic HLH (helix–loop–helix) transcription factors such as MyoD, myogenin, Mrf4, and Myf5 that are expressed downstream of Pax3 [13]. Figure 1 presents stages of myogenesis, showcasing the characteristic expression of transcription factors. During primary myogenesis, myoblasts stop growing, and they align and fuse to form multinucleated myotubes. This process continues into secondary myogenesis during the fetal stage, where more myotubes are created and surround the primary fibers. Muscle maturation continues in early postnatal development, resulting in muscle growth due to a hypertrophic response. Many review papers offer in-depth insights into the molecular basics of myogenesis [14]. Here, we focus on the role of NF-κB in regulating myogenesis during muscle development and regeneration.

### 2.2. NF-κB Family and Its Regulators

NF-κB is an evolutionarily conserved family of protein dimers encoded by five gene members RelA/p65, RelB, c-Rel, NF-κB1/p50 (from precursor p105), and NF-κB2/p52 (from precursor p100). These transcription factors exist as homodimers or heterodimers, which bind to classical κB sites or non-canonical sequences and act as positive regulators of transcription, excluding p50/p50 and p52/p52 homodimers, which lack transactivation domains [16]. Unlike typical transcription factors, NF-κB transcription factors do not initially reside in the nucleus but are held inactive in the cytoplasm under strict control of its inhibitory protein IκB (inhibitor of NF-κB). Upon activation signal, IκB undergoes phosphorylation, ubiquitination, and subsequent degradation, leading to the release of an NF-κB dimer that is subsequently translocated into nuclei; it also binds to its recognition sites on promotors, enhances sequences, and activates gene transcription. The family of IκB proteins that regulates NF-κB activity includes IκBα, IκBβ, IκBγ, IκBε, IκBζ, Bcl-3, and NF-κB precursor proteins, p100 and p105. IκB family members have a core of six or more ankyrin repeats that strongly bind with the Rel homology domain (RHD) in NF-κB. Upon binding, the nuclear localization signal (NLS) in NF-κB is hidden, preventing the NF-κB from entering the nucleus and keeping it in the cytoplasm of unstimulated cells [17]. The NF-κB pathway can be activated through two distinct signal pathways in mammals, each involving different activators, receptors, downstream elements, and NF-κB members [18]. The degradation of IκB proteins is controlled by the IκB kinase (IKK) complex, which is responsible for the phosphorylation of IκB. The IKK complex consists of two kinases, IKKα and IKKβ, along with oligomerized NEMO protein (NF-κB essential modulator), also called IKKγ. NEMO is required for the formation of a fully functional kinase complex. Various upstream signals activate NF-κB, including inflammatory cytokines, oxidative stress, mitogens, and bacterial products. The signals are directed through intracellular adapter proteins, enabling receptor-triggered signaling events that begin with IKK activation and end with NF-κB nuclear translocation. TNF receptor-associated factors (TRAFs) are adaptor protein family members, which are pivotal for NF-κB signaling pathways. The TRAFs serve as E3 ubiquitin ligases and are recruited to cell surface receptors by “classical” adaptor proteins like TRADD, MyD88, or IRAK. These interactions lead to the formation of multiprotein complexes that trigger the signal to the NF-κB pathway. The Receptor interaction proteins (RIPs) represent another family of “classical” adapter proteins that act as serine/threonine kinase that can bind to IKKγ, TRADD, and TRAFs. Activators of NF-κB include the IKK-related protein TBK1 (IKK complex-specific kinase). Phosphorylation of IKKβ leads to stimulation of IKKβ kinase activity towards IκB [19].

### 2.3. Canonical and Non-Canonical NF-κB Signaling

**Figure 2:**
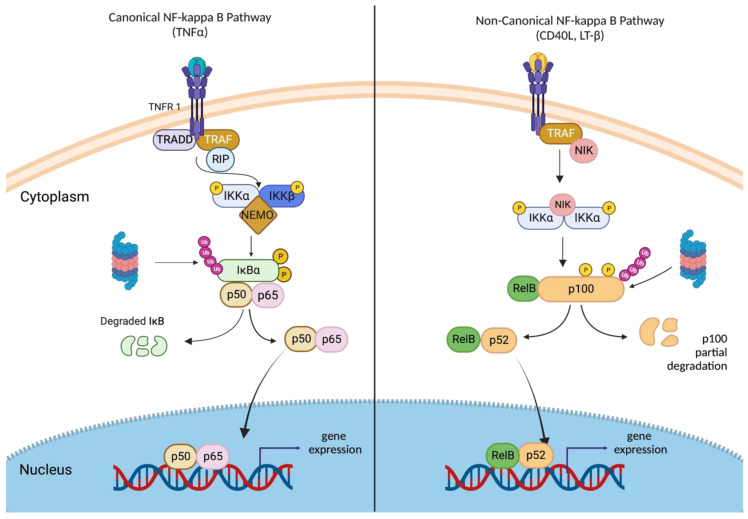
Canonical versus non-canonical activation of NF-κB pathway. Concisely, in the TNFα-signaling pathway (left), the activation of canonical NF-κB receptor TNFRs (tumor necrosis factor receptor) leads to the recruitment of TRADD (TNF receptor-associated death domain), TRAF, and RIP. Activation of RIP leads to the recruitment of NEMO (IKKγ)/ IKKβ /IKKα complex to receptor complex and causes IKKβ activation. Activated IKKβ phosphorylates IκBα that leads to its separation from the p65/p50 complex and degradation. Free p65/p50 heterodimers then translocate to the nucleus and regulate target gene transcription. In the non-canonical NF-κB-signaling pathway (right), activation of non-canonical NF-κB receptors, such as (LT)βR (lymphotoxin b–receptor), or CD40 (cluster of differentiation 40), triggers IKKα phosphorylation and activation by NIK. Activated IKKα then phosphorylates p100, leading to p100 polyubiquitination and partial processing to p52. p52/RelB heterotrimers translocate to the nucleus to regulate specific gene expression. Created with BioRender.com.

Two main pathways regulate NF-κB: the canonical (classical) and non-canonical (alternative) pathways (shown in Figure 2). These pathways are triggered by different stimuli, membrane receptors, adapter proteins, and NF-κB family members, and they are responsible for overseeing various cellular processes. The canonical pathway is activated by many extracellular and intracellular factors such as growth factors, proinflammatory cytokines, ROS (reactive oxygen species), DNA damaging agents, bacterial products, and viruses. Binding of ligands to NF-κB receptor TNFRs, TLRs, IL-1R, and the TCR and BCR trigger receptor-mediated assembly of the multiprotein-signaling complex, which leads to IKKγ and IKKβ activation, resulting in IκB degradation and the translocation of NF-κB (p65/p50) into nuclei [20].

The non-canonical, alternative NF-κB-signaling pathway is mediated by the IKKα homodimer complex. The binding of ligands (CD40, lymphotoxin β, and B cell-activating factor (BAFF)) to their receptors cause the stabilization of NF-κB-inducing kinase (NIK), which phosphorylates and activates IKKα homodimer. Activated IKKα then phosphorylates p100, leading to p100 polyubiquitination and partial processing to p52. Heterodimers—p52/RelB translocate to the nucleus to regulate specific gene expression [21].

### 2.4. Dual Role of NF-κB in Myogenesis

NF-κB pathways play a multifaceted and essential role in myogenesis. They regulate myoblast proliferation, survival, and MRF expression critical for myoblast differentiation [22]. Some studies have shown that NF-κB signaling also contributes to the inflammatory response and muscle regeneration after injury [23]. Numerous scientific reports have revealed that the activation of NF-κB is associated with the promotion of myogenesis. NF-κB pathways play a dual role in regulating myoblast proliferation and survival during myogenesis. Langen et al. showed that TNFα activation of the NF-κB pathway promotes myoblast proliferation by reducing stability and inducing the degradation of MyoD protein, thus inhibiting myoblast differentiation [24]. Additionally, NF-κB maintains myoblasts in a proliferative state by binding to the cyclin D1 promoter and directing its transcription, thereby stimulating the progression of the cells into the S phase. Guttridge et al. demonstrated that in myogenesis, this process is regulated via the classical p50/p65 heterodimer [25]. It was observed that NF-κB inhibits myogenesis via a different pathway by inducing the transcription factor YY-1, a component of the Polycomb repressive complex [26]. According to Bakkar et al. [27], the traditional NF-κB signaling decreases during differentiation, while the alternative members IKKα, RelB, and p52 are induced later during myogenesis. They showed that myogenesis was enhanced in myoblasts deficient in RelA/p65 or IKKβ, which are the components of the classical NF-κB signaling. Alternative signaling is crucial for maintaining myotubes in response to metabolic stress but is not required for myotube formation. Additionally, it has been observed that the overexpression or knockdown of IKKα can regulate mitochondrial content and function, indicating that the alternative signaling stimulates mitochondrial biogenesis. These findings suggest a unique IKK/NF-κB signaling switch that inhibits differentiation and promotes myotube homeostasis [27]. Furthermore, TWEAK (TNF-like weak inducer of apoptosis) interacts with Fn14 (fibroblast growth factor-inducible molecule 14) under physiological conditions, activating the non-canonical NF-κB pathway via TRAF adaptors. This, in turn, promotes myogenesis by increasing myoblast Fusion [28]. Gu et al. [29] reported that NF-κB signals in the NG2^+^ interstitial cells induce the expression of EphrinA5 to facilitate myoblast migration toward the end of growing myofibers and neonatal skeletal muscle growth [29].

### 2.5. NF-κB: Orchestrating Muscle Regeneration

Skeletal muscles in adults are tissue that remains stable and has minimal turnover. However, in case of injury or damage, muscle fibers undergo a regeneration process, primarily due to the existence of adult muscle stem cells—satellite cells.

Satellite cells are typically at rest and only occasionally divide through an asymmetric process to replace muscle fibers that have been damaged during daily activities. This process helps to maintain the pool of the stem cells [30]. The classical NF-κB pathway has been found to modulate muscle regeneration in response to damage and degenerative muscle disease. Straughn et al. [31] have shown that the canonical NF-κB pathway increases in satellite cells upon skeletal muscle injury. The authors reported that the physiological NF-κB signaling promotes the survival and proliferation of satellite cells. Furthermore, they revealed that IKKβ is essential for the successful regeneration of adult skeletal muscles [31]. The study of Schmidt et al. [32] has demonstrated that the stimulation of non-canonical NF-κB signaling using an agonistic LTβR antibody has detrimental effects on myogenic differentiation, muscle stem cell function, and the overall regeneration of skeletal muscle. Additionally, the study provided evidence that maintaining a delicate balance between the activation of the canonical and non-canonical signaling pathways is crucial for ensuring proper myogenic differentiation and optimal function of muscle stem cells [32].

The process of skeletal muscle myogenesis involves two distinct NF-κB-signaling pathways: canonical and non-canonical. The canonical pathway is crucial in keeping myoblasts in the proliferative stage and preventing premature differentiation. Once the canonical pathway is downregulated, the non-canonical NF-κB pathway is activated, promoting mitochondrial biogenesis and providing myotubes with the energy needed to synthesize myofibrillar proteins. Understanding the role of NF-κB in myogenesis is essential to develop new therapeutic compounds to treat skeletal muscle disorders.

### 2.6. The Role of NF-κB in Muscular Dystrophy

Muscular dystrophies encompass a group of hereditary muscular disorders, of which Duchenne muscular dystrophy (DMD) is the most common. DMD is an X-linked recessive condition characterized by the gradual degeneration of the muscle fiber membrane, primarily caused by a deficiency of the dystrophin sarcomeric protein [33]. Dystrophin, along with a complex of dystrophin glycoproteins, connects actin filaments to the cell membrane and participates in signal transduction within the cell. Its absence leads to a weakened cell membrane, enabling the influx of calcium ions, infiltration of immune cells, chronic inflammation, necrosis, and the subsequent degradation of muscle cells. In DMD, mutations result in a frameshift mutation and a complete absence of dystrophin in the muscles. Moreover, DMD patients experience cyclical episodes of muscle loss (degeneration) and regeneration. The secondary hallmark of DMD is that the microenvironment of dystrophic cells includes an increased number of inflammatory cells, acting as a complex interface for cytokine signaling [34]. In particular, elevated levels of tumor necrosis factor-α (TNF-α) are indicated. TNF-α is known to function as a robust stimulator of the transcription factor NF-κB, which plays a pivotal role in activating the inflammatory response. A wide range of key functions of NF-κB in skeletal muscles has been demonstrated. Zhou et al. have demonstrated that it acts as a crucial mediator of gene expression in response to redox signaling and actively participates in the activation of antioxidant enzymes, including glutathione peroxidase and catalase, in response to oxidative stress [35]. Monici et al. first demonstrated the activation of NF-κB in DMD, indicating its enhanced binding to DNA by electrophoretic mobility shift assay (EMSA) [36]. Currently, the chronic activity of NF-κB is known as a hallmark of this disease, showing its detrimental influence on skeletal muscle [37]. Moreover, it has been observed that the activation of NF-κB through the conventional pathway enhances myoblast proliferation (through activation of cyclin D1) and inhibits muscle regeneration (by interrupting MyoD expression) [25]. It has been discovered that the conventional NF-κB dimer composed of p50/p65 is triggered in *mdx* mouse (model of DMD) muscles, with p65 serving as the transcriptionally active subunit responsible for muscle pathology [38]. These observations align with studies conducted on individuals affected by DMD, implicating p65 as the principal NF-κB component involved in pathological muscle conditions [36].

Acharyya et al. described that the precise pharmacological inhibition of IKK resulted in decreased inflammation and, simultaneously, increased regeneration, implicating IKK/NF-κB as a suitable therapeutic target in DMD [39]. The authors underscored the essential role of NF-κB in the DMD’s progression. It has been shown that IKK/NF-κB signaling is persistently elevated in immune cells and regenerative muscle fibers in DMD mouse models and patients. This signaling pathway seems to promote chronic inflammation. Due to the diverse array of NF-κB target genes, including cytokines and chemokines, Acharyya et al. hypothesized that NF-κB-mediated transcription might serve as an amplifying signal for sustaining an enduring immune response within a dystrophic muscle. Moreover, the authors also described a second potential mechanism of action associated with the repression of muscle regeneration. The results obtained from their investigation propose that the inhibition of NF-κB activity, either through genetic manipulation or pharmacological intervention, facilitates the generation of novel myofibers in response to degenerative processes. It should also be noted that improvements in muscle pathology were observed in muscle-specific IKKβ-ablated mdx mice, indicating that NF-κB signaling in muscle cells plays a crucial role in muscle regeneration. In contrast, myeloid-specific IKKβ ablation reduced myonecrosis, suggesting that NF-κB signaling through inflammatory cells contributes to dystrophin deficiency pathology [39].

The disruption of the NF-κB-signaling pathway has also been observed in limb–girdle muscular dystrophy (LGMD), indicating a suppression of its protective function [40]. Limb–girdle Type 2A muscular dystrophy (LGMD2A) arises from mutations in the calpain-3 gene and manifests as muscle degeneration and weakness, primarily affecting the shoulder blade and pelvic muscles [41]. Calpain-3 is a skeletal muscle-specific cysteine protease that relies on calcium; its involvement in LGMD2A is still under investigation [42]. The occurrence of symptoms of programmed cell death in muscle nuclei, observed in both patient biopsies and mice lacking calpain 3, has been associated with altered NF-κB signaling [40]. NF-κB plays a vital role in promoting cell survival by regulating the transcription of factors that inhibit apoptosis [43]. However, one proposed hypothesis suggests that in LGMD2A, the build-up of IκBα within muscle nuclei hinders NF-κB signaling to genes involved in cell survival, such as cellular-FLICE inhibitory protein, ultimately leading to programmed cell death [40].

The treatment strategy for dystrophies has included corticosteroid agents, including prednisone, deflazacort, and alternative dosing patterns to reduce side effects. Even with the application of molecular-based medicines, this class of medication remains a crucial component of the treatment. Steroids like glucocorticoids are non-specific anti-inflammatory medications that act as NF-κB inhibitors [44]. Novel therapeutic approaches encompass the utilization of stop codon readthrough (ataluren) and exon skipping (eteplirsen, golodirsen, casimersen, viltolarsen) in the DMD gene. However, their application remains restricted to a subset of patients affected by DMD, which possesses specific detrimental variants in the DMD gene. Currently, gene replacement therapy exhibits promising outcomes, while the utilization of CRISPR/Cas9 holds tremendous potential, provided that the risks associated with off-target effects can be mitigated. Drugs targeting NF-κB and deflazacort as a substitute for prednisone have been developed for the same purpose. Those approaches have been precisely described by Mackenzie et al. [44].

The data described above indicate the complex nature of the perturbation of NF-κB signaling in muscular dystrophy, exerting effects on both inflammatory processes and the functionality of muscle cells. Unfortunately, to date, knowledge of the mechanisms of action or regulation of NF-κB signaling in muscular dystrophy is scarce.

### 2.7. NF-κB in Myositis

Myositis is caused by an abnormal immune response, which leads to muscle damage [45]. Unless inflammation is the result of an infection or other systemic diseases such as cancer, amyloidosis, or sarcoidosis, the cause of myositis is often unknown [46]. Idiopathic inflammatory myopathies can be subdivided into polymyositis, dermatomyositis, and inclusion body myositis. Regardless of etiology, all inflammatory myopathies share several common characteristics: they cause weakness and decreased muscle performance; changes in muscle mass; and they show a presence of inflammatory infiltrates or even necrosis due to the prolonged activity of the immune system [45].

NF-κB plays a significant role in the development and progression of myositis, particularly in autoimmune forms of the condition [46]. In myositis, the persistently increased activity of NF-κB during inflammation is the result of a disturbed negative feedback loop, the self-sustained inflammatory response. Toll-like receptors (TLRs), particularly TLR3, TLR7, and TLR4, are also involved in myositis pathogenesis. The binding of specific antigens to these receptors leads to the direct activation of the transcription factor NF-κB. The entire process is influenced not only by pro-inflammatory cytokines or TNF-type chemokines but also by oxidative, mechanical, and endoplasmic reticulum (ER) stress [47]. These factors cause the appearance of other stimulants in the environment, such as damage-associated molecular patterns (DAMPs) or enzymes associated with damaged fibers, such as creatine kinase and other ligands involved in the NF-κB activation pathway and transcription of cytokine genes. NF-κB promotes fibrosis and excessive accumulation of connective tissue, leading to muscle stiffness and reduced muscle function. Wu et al. have shown that even low levels of this factor have a profibrogenic effect, stimulating the production of fibrotic factors such as cytokines (IL-13, IL-21, TGF-β1), chemokines (MCP-1, MIP-1β), and peroxisome proliferator-activated receptors (PPARs) [48]. Immunohistopathological studies show that patients diagnosed with idiopathic myositis have elevated levels of NF-κB subunits, particularly active p65 [49]. One reason for the deposition of the factor subunits is the accumulation of amyloid beta proteins and phosphorylated tau, a main activator of NF-κB [9]. Muscle fibrosis is also accompanied by an abnormal atrophic fiber structure. It has been proven that NF-κB also affects pathological rearrangement by activating protein degradation through ubiquitin-dependent proteolysis [9]. An attempt to repair muscle tissue results in the overexpression of HLA class antigens and tool-like receptors (TLRs) causes an even greater sensitization of the tissue to inflammatory cytokines and other chemokines, especially since in these conditions they are present not only on the cells of the immune system but also on the muscle fibers, causing their further degradation [50]. Additionally, the activation of NF-κB impairs myoblast differentiation and further growth and development by blocking the transcription factor MyoD, which causes dystrophy [51].

In addition to deposits and fibrosis, the characteristic clinical picture of myositis is the presence of lymphocytic infiltrates, mainly CD4/CD8 T-cells [52]. In myopathies, these cells contain both NF-κB p65/p50 and I-κBα subunits [53]. Their presence has also been confirmed in other immune system cells, such as CD20+ B cells and CD68+ macrophages, on the membranes of dendritic cells. The presence of factor subunits, apart from the main task of inducing the production of cytokines, also causes an increased cell recruitment and the formation of cell infiltrates. Carrero et al. have demonstrated that IL-1β activates NF-κB, resulting in the transcriptional activation of a wide range of genes, such as adhesion molecule mediators and growth factors, which are often chemotactic for other cells, such as neutrophils and monocytes [54]. An example of such a molecule is MCP, an NF-κB-regulated chemoattractant that enables the active invasion of immune cells deep into muscle fibers [55]. The NF-κB-signaling pathway maintains a balance between cell survival and apoptosis in normal cells. An accurate understanding of the mechanisms of action of NF-κB in the pathogenesis of myositis and related diseases will be the first step in developing new therapies based not only on acute treatment but also on causal treatment before irreversible damage and changes in the form of infiltrates occur. The solution may be antibodies that block the activity of NF-κB itself or other participants in signaling pathways closely related to this transcription factor, such as TLRs. Another way to achieve a therapeutic effect may be to use inhibitors or antagonists of receptors in mTOR/NF-κB signaling [56]. While initial research has shown promising results, this topic must be explored more thoroughly.

## 3. Substances of Natural Origin Targeting NF-κB

Substances of natural origin are compounds that have been intensively analyzed in recent years. They are obtained, among others, from plants, fungi, arthropods, higher vertebrates, marine invertebrates, and secretions of microorganisms. Natural products have numerous anti-cancer effects, such as pro-apoptotic, anti-proliferative, and anti-metastatic, and they also regulate autophagy and balance immunity [57]. In the following subsections, we will discuss various substances of natural origin that affect the expression of NF-κB.

### 3.1. Curcumin

Curcumin is a polyphenol compound found in the spice turmeric *(Curcuma longa)*. It has been shown to inhibit NF-κB signaling in various cancer types, including rhabdomyosarcoma [58]. Curcumin, as a natural chemotherapeutic drug, can inhibit the initiation and progression of carcinogenesis. It is also characterized by antioxidant and anti-inflammatory effects [59]. This substance has inhibitory properties against factors responsible, among others, for the proliferation or inhibition of apoptosis, e.g., COX-2, AP-1, or NF-κB, probably by inhibiting IκB phosphorylation [60]. It works by inhibiting the activation of the IKK complex, which is responsible for NF-κB activation [61]. In addition, studies using tenocytes have shown that curcumin degrades the nuclear factor IκBα and inhibits the translocation of p65 to the nucleus [62]. In addition, curcumin exhibits anti-cancer effects inducing apoptosis by reducing the level of NF-κB, IL-6, and IL-11 while modulating AMPK, AKT/mTOR, STAT, and p53 signaling [63,64].

### 3.2. Epigallocatechin-3-Gallate (EGCG)

Epigallocatechin-3-gallate (EGCG) is an active chemical compound from the group of flavonoids found abundantly in green tea. It has been analyzed and attributed to its antioxidant, anti-inflammatory, and anti-cancer properties [65]. It is cytotoxic to rhabdomyosarcoma cells by reducing cell proliferation [66]. Ahmad et al. demonstrated that EGCG treatment resulted in cell growth inhibition, cell cycle G0/G1 arrest, and apoptosis induction in human epidermal carcinoma cells (A431) but not in normal human epidermal keratinocytes (NHEK). Cell cycle deregulation and apoptosis of tumor cells induced by EGCG probably occur due to the inhibition of the NF-κB factor [67]. Additionally, EGCG has been shown to act as a pro-oxidant by generating reactive oxygen species and reducing NF-kB levels [68]. EGCG also blocks LPS-induced IκBα degradation, the translocation of RelA into the nucleus, and the DNA-binding activity of NF-κB [69]. Additionally, EGCG exhibits anti-cancer effects by inhibiting cell proliferation by upregulating miR-1and inhibiting the activation of the IGF-1R pathway [70,71].

### 3.3. Resveratrol

Resveratrol is the substance that is most abundant in red grapes, mainly in the seeds and skin. It is also found in peanuts and black currants. It has anti-inflammatory, anti-cancer, anti-diabetic, and antioxidant properties, and it also participates in the regulation of the NF-κB-signaling pathway [72,73]. Resveratrol works by inhibiting the activation of the IκB kinase (IKK) complex, which is responsible for NF-κB activation [70]. This natural substance has been proven to act as an inhibitor of COX-2 activity and block TPA-induced NF-κB activation and COX-2 expression in mouse skin in vivo [71]. Resveratrol inhibits NF-κB activation in a dose-dependent manner. This substance reduces the transcriptional activity of p65 but did not affect the DNA-binding activity of NF-kκB or block p65 nuclear translocation [69]. Additionally, resveratrol exhibits anti-cancer activity by stopping the S/G2 phase of the cell cycle in cells, reducing the expression of cyclin B, inhibiting cell growth by engaging the AKT and caspase-3 pathways, inhibiting cell migration, and reducing the level of IL-8 secretion [71,72].

### 3.4. Quercetin

Quercetin is a flavonoid found mainly in onions, grapes, broccoli, and citrus fruits. It has antioxidant, anti-cancer, and anti-inflammatory properties and is used to prevent cardiovascular diseases [73]. The action of quercetin affects the NF-κB factor in several ways. It inhibits I-κB phosphorylation, stops NF-κB translocation into the cell nucleus, and prevents NF-κB-DNA binding and reporter gene transcription [74]. Quercetin has been shown to promote apoptosis of cancer cells by regulating the NF-κB factor and Bcl-2 and Bax genes [75]. Additionally, quercetin exhibits anti-cancer activity by binding the NR4A1 protein and inhibiting NR4A1-dependent transactivation in cells. It weakens the migration and invasion of cells and has the ability to reduce the levels of mRNA and proteins HIF-1α, VEGF, MMP2, and MMP9. It reduces the ROS formation [75,76,77,78].

### 3.5. Berberine

Berberine is a compound belonging to the group of isoquinoline alkaloids. It occurs naturally in many plants, e.g., from the genera *Annonaceae*, *Berberidaceae*, or *Menispermaceae*. It has been shown that this substance has antioxidant properties, affects the contractility of the heart, and has anti-cancer effects, also on rhabdomyosarcoma [79,80]. The action of berberine consists in inhibiting the phosphorylation of the nuclear factor IκBα responsible for the activation of NF-κB. In addition, it has been proven that after treatment with berberine, the phosphorylation of the p65 transcription factor is inhibited [81]. Berberine exhibits anti-cancer activity by downregulating caspase-1/IL-1β [82]. In addition, in RMS cells, it inhibits the cell cycle in the G1 phase [76].

### 3.6. Caffeic Acid Phenethyl Ester (CAPE)

Caffeic acid phenethyl ester (CAPE) is one of the components found in propolis. Numerous scientific reports indicate that CAPE has antioxidant, anti-inflammatory, and cytotoxic effects on cancer cells. One of the proposed mechanisms of anticancer activity is the modulation of the NF-κB-signaling pathway [83]. It has been shown that CAPE can inhibit NF-κB signaling by preventing the activation of upstream kinases, such as IκB kinase (IKK) and TGF-β-activated kinase (TAK1), which are involved in the activation of the NF-κB pathway [84]. Caffeic acid has also been shown to inhibit the nuclear translocation of NF-κB and its binding to DNA, which are essential steps in the transcriptional activation of NF-κB target genes [85]. In addition to inhibiting NF-κB signaling, caffeic acid has been reported to activate the Nrf2-signaling pathway, which plays a key role in cellular defense against oxidative stress and inflammation. The activation of Nrf2 can lead to the upregulation of antioxidant enzymes, such as heme oxygenase-1 (HO-1), which can protect cells from oxidative damage and inflammation [86]. Additionally, CAPE in fibrosarcoma cells has been shown to increase ROS levels leading to lipid peroxidation, which in turn alters the mitochondrial membrane potential. It also causes oxidative DNA damage [87].

### 3.7. Cucurbitacin E (CurE)

Cucurbitacin E (CurE) is a triterpenoid compound found in various plants, including bitter melon (*Momordica charantia*) and cucumber (*Cucumis sativus*) [88]. Cucurbitacin E has been shown to have anti-inflammatory and anticancer properties by inhibiting proliferation and inducing apoptosis, and one proposed mechanism of action is the modulation of the NF-κB-signaling pathway [89]. The compound has been shown to modulate NF-κB signaling by inhibiting its activation and nuclear translocation [90]. Cucurbitacin E has been shown to inhibit TNF- α induced phosphorylation of inflammatory cytokines by modulating the NF-κB pathway [91]. In addition, cucurbitacin E inhibits the PI3K/Akt/mTOR pathway and the epithelial-mesenchymal transition (EMT) in osteosarcoma cells [92].

The Table 1 below provides a comprehensive overview of the method of action for various substances of natural origin against muscle-derived cancers, both in vitro and in vivo. 

## 4. Conclusions

NF-κB is an inducible transcription factor that mediates the expressions of a wide array of genes associated with various immune and inflammatory responses. A huge amount of data from scientific publications suggests the involvement of the NF-κB factor in the formation and progression of various cancers, including those of muscle origin. That is why it is so important to recognize changes in cancer cells that cause, among others, the overexpression of the NF-κB factor. The involvement of NF-κB in the induction and proliferation of cancer cells via metastasis, angiogenesis, the regulation of growth signals, and the inhibition of apoptosis makes it a suitable therapeutic target against cancer. In this review, we have highlighted naturally occurring substances that have been proven to regulate NF-κB expression and discussed their mechanism of inhibition, which can be further explored as a potential future anti-cancer therapy. These substances are natural compounds that have advantages over existing synthetic NF-κB inhibitors in terms of safety and effectiveness. The use of these substances as adjuvant chemotherapy, together with conventional cancer treatment, may represent an improved therapeutic strategy. However, regarding the interaction of these natural compounds with standard therapies for muscle-derived cancers, such as chemotherapy or radiation therapy, it is important to note that the potential interactions between natural compounds and conventional cancer treatments can be complex and multifaceted. The effects of these compounds may vary depending on the specific cancer type, stage, treatment regimen, and individual patient factors. To determine the impact of natural compounds on standard cancer therapies, rigorous clinical trials are needed to evaluate the safety, efficacy, and potential interactions.

## Figures and Tables

**Table 1:** The method of action of selected substances of natural origin in the muscle-derived cancers in vitro or in vivo.

Compound	Cancer	Stimulated Pathway	References
Curcumin	Fibrosarcoma	Inducing apoptosis by downregulating NF-κB, IL-6, and IL-11.	[57,59,60,61,93,94]
	Osteosarcoma	Apoptosis suppresses the proliferation, invasion, and metastasis.	
	Rhabdomyosarcoma	Suppressing NF-κB activity, modulation of AMPK, AKT/mTOR, STAT, and p53 signaling.	
Resveratrol	Rhabdomyosarcoma	Inhibition of cell proliferation, induces arrest of the S/G2 phase of the cell cycle and reduction of cyclin B expression.	[95,96,97]
	Fibrosarcoma	Induces apoptosis, inhibition of cell proliferation, expression of apoptosis-associated genes was altered.	
	Osteosarcoma	Inhibition of cell growth through the involvement of the AKT and caspase-3 pathways, inhibition of cell migration and a decrease in the level of IL-8 secretion.	
Epigallocatechin-3-gallate (EGCG)	Rhabdomyosarcoma	Reducing cell proliferation and downregulating the HH signaling pathway.	[64,65,66,67,68,98]
	Osteosarcoma	Inhibition of cell proliferation by upregulation of miR-1.	
	Ewing sarcoma	Induction of apoptosis, inhibition of cell proliferation, and inhibition of activation of the IGF-1R pathway.	
Quercetin	Rhabdomyosarcoma	NR4A1 protein binding and inhibition of NR4A1-dependent transactivation in cells, regulating the pro-oncogenic genes PAX3-FOXO1 and G9a.	[76,77,78]
	Fibrosarcoma	Inhibition of cell motility by inhibiting the activation of MMPs, attenuating the formation of ROS.	
	Osteosarcoma	Attenuation of cell migration and invasion, downregulation of expression levels of mRNA and proteins HIF-1α, VEGF, MMP2, and MMP9.	
Berberine	Rhabdomyosarcoma	Inhibited the cell cycle of all RMS cells at the G1 phase.	[88,93]
	Osteosarcoma	Downregulating the caspase-1/IL-1β.	
Caffeic acid phenethyl ester (CAPE)	Fibrosarcoma	Enhances the ROS levels and lipid peroxidation alters the mitochondrial membrane potential increased oxidative DNA damage, apoptosis.	[87]
Cucurbitacin E (CurE)	Osteosarcoma	Inhibited the PI3K/Akt/mTOR pathway and epithelial-mesenchymal transition (EMT).	[92]

## Data Availability

Not applicable.
